# Findings from a Malaysian multicentre study on oropharyngeal squamous cell carcinoma

**DOI:** 10.1186/s13027-023-00557-0

**Published:** 2023-11-28

**Authors:** Hans Prakash Sathasivam, Sangeetha Passu Davan, Szu May Chua, Rahmuna Fazlina Rohaizat, Rohaizam Japar, Zahirrudin Zakaria, Abd Razak Ahmad, Hasmah Hashim, Shashi Gopalan Marimuthu, Yew Toong Liew, Doh Jeing Yong, Pappathy Vairavan, Avatar Singh Mohan Singh, Benjamin Hong Beng Goh, Zulkifli Yusof, Khairul Azlan Shahril Abu Dahari, Ali Haron, Masaany Mansor, Mohd Zambri Ibrahim, Shiraz Qamil Muhammad Abdul Kadar, Mohamad Hazri Hamal, Wan Emelda Wan Mohamad, Malaysian Head & Neck Cancer Research Group

**Affiliations:** 1 Institute for Medical Research, National Institutes of Health, Ministry of Health Malaysia, Setia Alam, Malaysia; 2 Hospital Kuala Lumpur, Ministry of Health Malaysia, Kuala Lumpur, Malaysia; 3 Hospital Pulau Pinang, Ministry of Health Malaysia, Pulau Pinang, Malaysia; 4 Hospital Melaka, Ministry of Health Malaysia, Melaka, Malaysia; 5 Hospital Tengku Ampuan Rahimah, Ministry of Health Malaysia, Klang, Malaysia; 6 University Malaya, Kuala Lumpur, Malaysia; 7 Hospital Queen Elizabeth, Ministry of Health Malaysia, Kota Kinabalu, Malaysia; 8 Hospital Sultan Ismail, Ministry of Health Malaysia, Johor Bahru, Malaysia; 9 Hospital Taiping, Ministry of Health Malaysia, Taiping, Malaysia; 10 Hospital Raja Permaisuri Bainun, Ministry of Health Malaysia, Ipoh, Malaysia; 11 Hospital Sultanah Bahiyah, Ministry of Health Malaysia, Alor Setar, Malaysia; 12 Hospital Tuanku Jaafar, Ministry of Health Malaysia, Seremban, Malaysia; 13 Hospital Raja Perempuan Zainab II, Ministry of Health Malaysia, Kota Bharu, Malaysia; 14 Universiti Teknologi MARA, Selangor, Malaysia; 15 Hospital Tuanku Fauziah, Ministry of Health Malaysia, Kangar, Malaysia; 16 Hospital Sungai Buloh, Ministry of Health Malaysia, Sungai Buloh, Malaysia; 17 Hospital Ampang, Ministry of Health Malaysia, Ampang, Malaysia; 18 Hospital Tengku Ampuan Afzan, Ministry of Health Malaysia, Kuantan, Malaysia

**Keywords:** Oropharyngeal, Oropharyngeal squamous cell carcinoma, Human papillomavirus, p16

## Abstract

**Background:**

In addition to the conventional aetiologic agents of oropharyngeal squamous cell carcinoma (OPSCC) such as tobacco usage, alcohol consumption and betel quid usage, it has been established that a proportion of OPSCC are driven by persistent oncogenic human papillomavirus (HPV) infections. Currently, there is a lack of data on the burden of HPV- associated OPSCC in Asian countries including Malaysia.

**Methods:**

A cross-sectional multicentre study with tissue analysis of Malaysian patients diagnosed with primary OPSCC within a five-year period, from 2015 to 2019 between 01/01/2015 to 31/12/2019 was undertaken. Determination of HPV status was carried out using p16INK4a immunohistochemistry on tissue microarrays constructed from archived formalin-fixed paraffin-embedded tissue.

**Results:**

From the cases identified, 184 cases had sufficient tissue material for analysis. Overall, median age at diagnosis was 63.0 years (IQR = 15) and 76.1% of patients were males. In our cohort, 35.3% of patients were Indian, 34.2% were Chinese, 21.2% were Malay and 9.2% were from other ethnicities. The estimated prevalence of HPV-associated OPSCC in our cohort was 31.0% (CI 24.4–38.2%). The median age for the HPV-associated OPSCC sub-group of patients was not significantly lower than the median age of patients with HPV-independent OPSCC. More than half of HPV-associated OPSCC was seen in patients of Chinese ethnicity (54.4%). Patients with HPV-associated OPSCC had a much better overall survival than patients with HPV-independent OPSCC (Log rank test; *p* < 0.001). Patients with HPV-associated OPSCC with no habit-related risk factors such as smoking, were found to have much better overall survival when compared to all other sub-groups.

**Conclusions:**

The findings from our study suggests that prevalence of HPV-associated OPSCC in Malaysia, though not as high as some developed countries, is however on an upward trend. HPV-associated OPSCC appears to be more frequently encountered in patients of Chinese ethnicity. Conventional risk-factors associated with OPSCC such as smoking, alcohol consumption and betel quid chewing should still be considered when estimating prognosis of patients with HPV-associated OPSCC.

## Background

The recent Global Cancer Observatory (GLOBOCAN) 2020 report estimated that more than 19 million new cases of cancer were diagnosed worldwide, with cancer being the leading cause of death in approximately 57 countries [1, 2]. Head and neck cancers are comprised of a diverse group of malignancies involving the various sub-sites of the head and neck region. In 2020, it was estimated that head and neck cancers (including lip, oral cavity, nasopharynx, larynx, hypopharynx and oropharynx) accounted for approximately 878, 348 new cancer cases (4.6%), making this group of cancers one of the top ten cancers in the world [1]. Oropharyngeal cancer is one of the cancers that has been showing an increasing trend with regard to incidence. There were approximately 98.412 new cases of oropharyngeal cancers in the 2020 GLOBOCAN report, an increase of more than 5000 from the previous 2018 GLOBOCAN report. [1, 3] Interestingly, the number of new deaths resulting from oropharyngeal cancers was lower in the 2020 GLOBOCAN report when compared to the 2018 GLOBOCAN report [1, 3].

The majority of oropharyngeal cancers are squamous cell carcinomas (SCC). In addition to the conventional aetiologic agents of oropharyngeal squamous cell carcinoma (OPSCC) such as tobacco usage, alcohol consumption and betel quid usage, it has been proven that a high proportion of OPSCC are driven by oncogenic human papillomavirus (HPV) [4]. HPV are DNA viruses that are known to infect mucosal and cutaneous epithelium, with persistent infection being known to cause benign or malignant tumours. Of the more than 100 HPV genotypes, the International Agency for Research on Cancer (IARC) has classified several genotypes as being “high-risk” genotypes and some as “low-risk” genotypes [5]. The IARC has classified HPV genotypes 16, 18, 31, 33, 35, 39, 45, 51, 52, 56, 58 and 59 as carcinogenic, HPV68 as “probably carcinogenic to humans” and HPV genotypes 26, 53, 66, 67, 70, 73, 82, 30, 34, 69, 85, 97 as “possibly carcinogenic” [5]. HPV genotypes 6 and 11, are considered as “low-risk” genotypes and have been known to cause anogenital warts [5, 6].

It has been estimated that approximately 690,000 cases of cancers worldwide in 2018 were attributable to HPV [7]. The vast majority of these cancers were cervical cancers (approximately 570,000 cases), followed by OPSCC (approximately 42,000 cases), anal SCC (approximately 29,000 cases), penile carcinoma (approximately 18,000 cases), and other sites making up the remaining cases [7]. Epidemiological studies have revealed that HPV16 and HPV18 genotypes are implicated in more than 70% of all cancers attributable to HPV [7]. Additionally, it is believed that the large majority (80–90%) of HPV-associated OPSCC is due to HPV16 [4, 8]. It has been hypothesized that the increase in HPV-associated OPSCC could possibly be due to changes in sexual practices and behaviour over the past few decades [8].

Patients with HPV-associated OPSCC have been shown consistently to have better survival than patients with HPV-independent OPSCC prompting the creation of distinct staging algorithm for patients with HPV-associated OPSCC [9]. However, much of the existing data regarding the prevalence, clinico-pathological features, and clinical outcome of patients with HPV-associated OPSCC has been derived from Caucasian populations [4]. The prevalence of HPV-associated OPSCC as well as clinical features of patients with this disease is not well characterized among Asian patients. At this point in time, there has only been one study looking into the prevalence of HPV- associated OPSCC in Malaysia. The study described the clinico-pathological features of HPV-associated OPSCC in a relatively small cohort of 60 patients with incomplete data that may not have been truly representative of the Malaysian population [10]. The current study was undertaken to address this gap in knowledge and to provide much needed data to inform policy making.

## Methods

### Patients

This was a cross-sectional multi-centre study, with tissue analysis, of Malaysian patients diagnosed with primary OPSCC within a five-year period, from 2015 to 2019. This study was performed with ethical approval from the Malaysian Medical Research and Ethics Committee (NMRR-20-49-52574) and complies with local regulations and guidelines. Individual patient consent was not sought as patients were not recruited for the study. Anonymised, archived tissue samples were analysed. The study was performed in accordance with the Declaration of Helsinki. Cases were identified from clinical and pathology databases. One representative formalin-fixed paraffin-embedded (FFPE) block, either from biopsy or resection specimen, with adequate tissue for immunohistochemistry (IHC) was selected for each patient. Cases were excluded if there was inadequate FFPE tissue for IHC. The following data were obtained from the archived records: sex; age at diagnosis; ethnicity; history of tobacco use; history of alcohol intake; history of betel-quid use; site of tumour; Tumour, Node, Metastasis (TNM) staging and date of last follow-up or death. Overall mortality status was obtained until end of June 2022. Data were link-anonymised and recorded into a standardised proforma. Determination of HPV status for each case was through assessment of surrogate marker p16^INK4a^ IHC (70% nuclear and cytoplasmic cut-off) [9, 11, 12]. Cases were considered as HPV-associated OPSCC if p16^INK4a^ IHC was scored as being positive and HPV-independent OPSCC if p16^INK4a^ IHC was scored as being negative.

### Histopathological assessment

Fresh 5 µm sections were taken from each FFPE block and mounted onto microscope slides. Haematoxylin and eosin (H&E) staining was performed on the Leica ST5010 Autostainer XL (Leica Biosystems, USA). Histopathological diagnosis was confirmed through review of the H&E-stained slides by the study pathologists and areas of confluent tumour within the tissue specimens were identified for construction of tissue microarrays (TMAs) for p16^INK4a^ IHC. For cases where TMA construction was not possible (tissue too small or unable to obtain tissue blocks), whole sections were used for p16^INK4a^ IHC. Tissue microarrays were constructed following previously described methods [13]. Haematoxylin and eosin–stained sections of the TMAs were made and assessed to confirm sampling accuracy.

### p16^INK4a^ immunohistochemistry (IHC)

Immunohistochemistry was performed using a monoclonal p16^INK4a^ antibody (JC2 clone; Cell Marque, Rocklin, USA). Five-micron sections of formalin-fixed paraffin-embedded (FFPE) tissues were manually stained by using a proprietary kit (EnVision FLEX+, Dako, Agilent Technologies, Santa Clara, California USA). The HPV/p16 Analyte Control block (HistoCyte Laboratories Ltd, Newcastle upon Tyne, UK) which demonstrates high homogenous, high heterogenous and negative expression of p16 was used for positive and negative control. Scoring for p16^INK4a^ IHC were done according to previously published criteria [11]. Briefly, p16^INK4a^ IHC was scored as being positive only if there was strong and diffuse nuclear and cytoplasmic staining present in greater than 70% of the tumour cells [9, 11, 12].

### Statistics

Statistical analysis was performed using IBM SPSS for Windows version 26 (IBM Corp., Armonk, N.Y., USA). Parametric and non-parametric tests were used for initial analysis of variables. Time-to-event analysis was performed to assess the influence of HPV status on overall survival using the Log Rank test. Statistical significance was defined at the 5% level.

## Results

From the cases identified, 184 cases had sufficient formalin-fixed paraffin-embedded tissue material for analysis. Overall, median age at diagnosis was 63.0 years (IQR = 15) and 76.1% of patients were males. In our cohort, 35.3% of patients were Indian, 34.2% were Chinese, 21.2% were Malay and 9.2% were from other ethnicities. The tonsils were the most frequently encountered sub-site for OPSCC (41.8%) followed by base of tongue tumours (30.4%). Smoking history was only available for 146 patients, with 59.6% of these patients having a positive smoking history (either current or former smokers). The majority of OPSCC patients presented with high-stage disease (Stage III or IV) when TNM staging was based on the 7th Edition of American Joint Committee on Cancer (AJCC) Staging system. Demographic and clinico-pathological features of these patients stratified according to p16 status are displayed in Table 1.

**Table 1:** Demographic and clinical characteristics (n = 184)

Characteristics		HPV-associated OPSCC (n = 57)	HPV-independent OPSCC (n = 127)	*p* value
Age Median [IQR] (Range)		63.0 [11] (26–78)	64.0 [15] (26–90)	0.344^Ϯ^
Sex	Male	46	94	0.356*
	Female	11	33	
Ethnicity	Malay	12	27	< 0.001**
	Chinese	31	32	
	Indian	8	57	
	Others	6	11	
^a^History of tobacco use	No	19	40	1.000*
	Yes (Current/Former)	27	60	
	Not available	11	27	
^a^History of alcohol intake	No	29	59	1.000*
	Yes (Current/Former)	14	29	
	Not available	14	39	
^a^History of betel quid use	No	35	59	< 0.001*
	Yes (Current/Former)	0	23	
	Not available	22	45	
Site of tumour	Tonsil	33	44	0.002**
	Base of tongue	13	43	
	Soft palate & uvula	9	15	
	Oropharynx Not Otherwise Specified (NOS)	2	25	
TNM staging Based on 7th edition of AJCC staging	Stage I	0	3	0.789**
	Stage II	3	8	
	Stage III	11	22	
	Stage IVA	31	58	
	Stage IVB	7	18	
	Stage IVC	5	18	

^Ϯ^Independent t-test; *Fisher’s exact test; ** Fisher–Freeman–Halton Exact Test; IQR = interquartile range; ^a^ Statistical test excluded cases with unavailable data

In our study, 57 patients were found to have p16^INK4a^ IHC positivity, giving an estimated prevalence of 31.0% (95% confidence interval of 24.4 to 38.2%) for HPV-associated OPSCC in our cohort. The median age for the HPV-associated OPSCC sub-group of patients was slightly lower at 63.0 years (IQR = 11), this however was not statistically significant. The majority of HPV-associated OPSCC was seen in Chinese patients (54.4%) whilst the majority of HPV-independent OPSCC was seen in Indian patients (44.9%) (Table 1; Fisher-Freeman-Halton Exact Test; *p* < 0.001). The tonsils were the most frequently site for HPV-associated OPSCC (57.9%) (Table 1; Fisher-Freeman-Halton Exact Test; *p* = 0.002).

Sub-analysis of cases with available habit related history showed the following results; smoking history and alcohol consumption history were not statistically associated with HPV status of OPSCC whilst history of betel quid usage was significantly associated with HPV status of OPSCC (Fisher’s exact test; *p* << 0.001). None of the HPV-associated OPSCC patients had a history of betel quid usage.

**Fig. 1:**
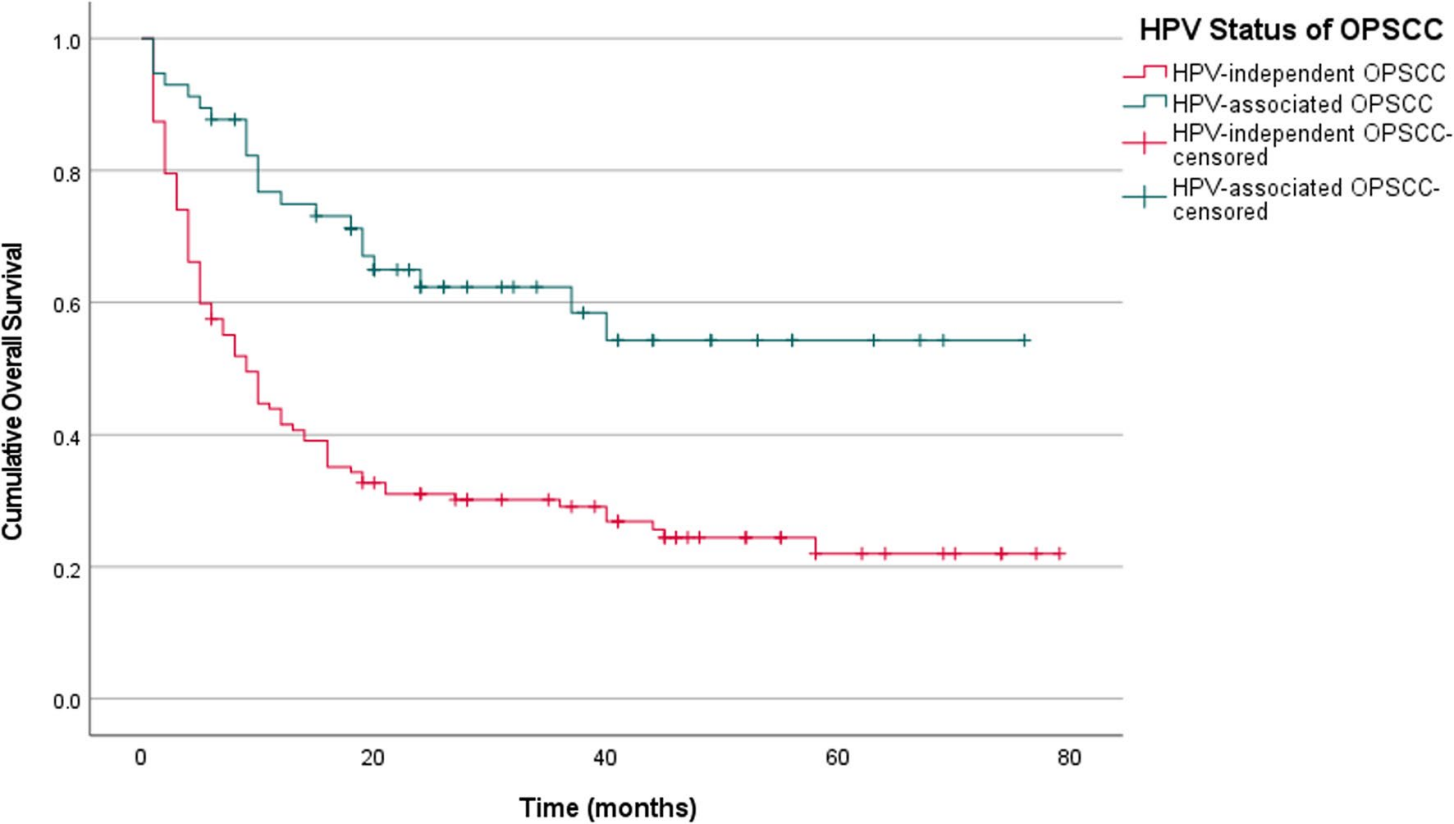
Kaplan Meier estimates of overall survival stratified according to HPV status. Patients with HPV-associated OPSCC appear to have much better overall survival rates when compared to patients with HPV-independent OPSCC (Log-rank test; *p* < 0.001; n = 184)

**Fig. 2:**
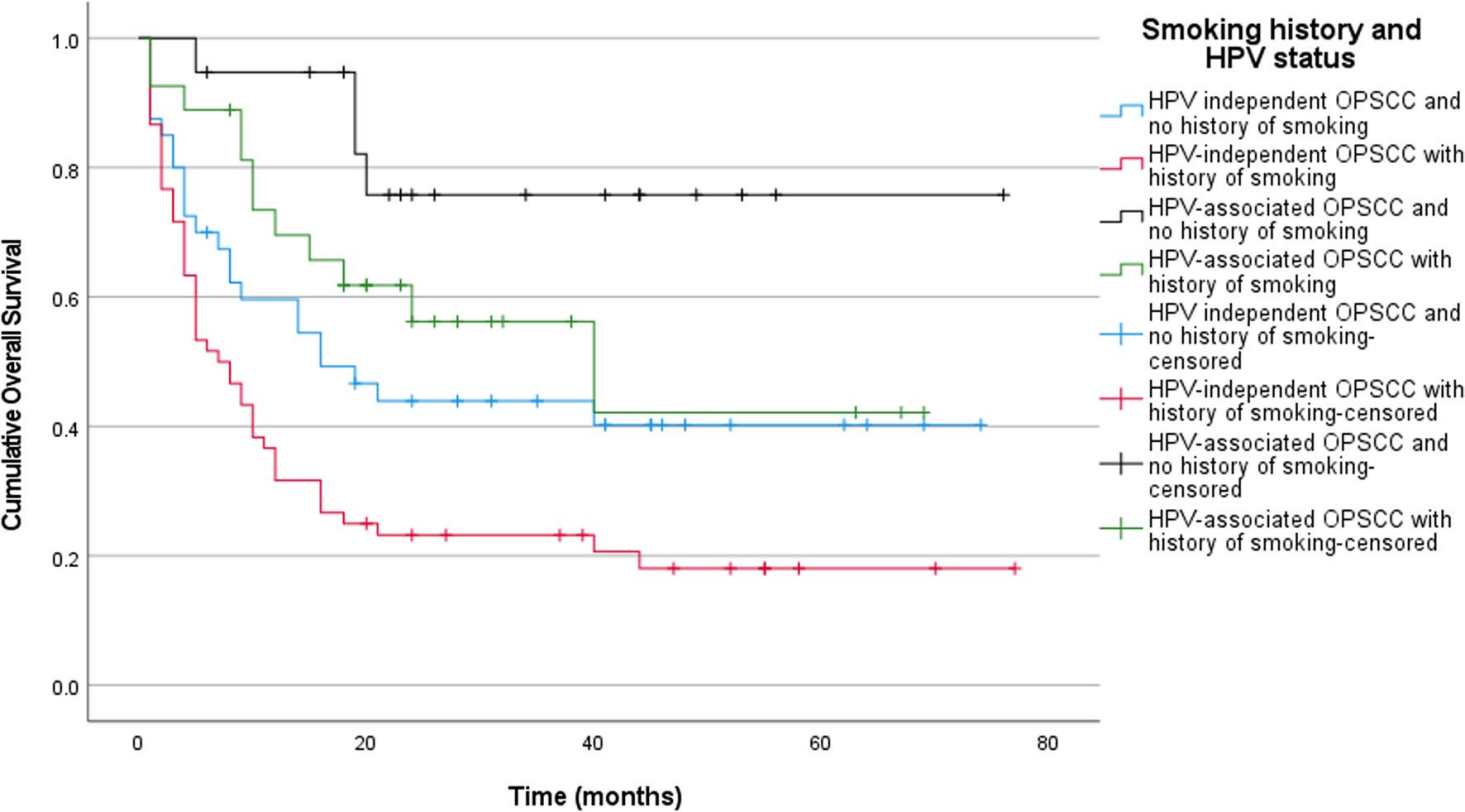
Kaplan Meier estimates of overall survival stratified according to smoking status in combination with HPV status. Never smokers with HPV-associated OPSCC had the best overall survival rates compared to other sub-groups (Log-rank test, pooled over strata; *p* < 0.001; n = 146). Pairwise statistical comparisons between patients with HPV-independent OPSCC who had no history of smoking and those that did, showed statistically significant difference between the two (Log-rank test, pairwise over strata; *p* = 0.021; n = 146). However, pairwise statistical comparisons between patients with HPV-associated OPSCC who had no history of smoking and those that did, showed no statistically significant difference (Log-rank test, pairwise over strata; *p* = 0.074; n = 146). Interestingly, pairwise statistical comparisons between patients with HPV-associated OPSCC with history of smoking and non-smokers with HPV-independent OPSCC had a rather similar prognosis with no statistically significant difference between them (Log-rank test, pairwise over strata; *p* = 0.286; n = 146)

**Fig. 3:**
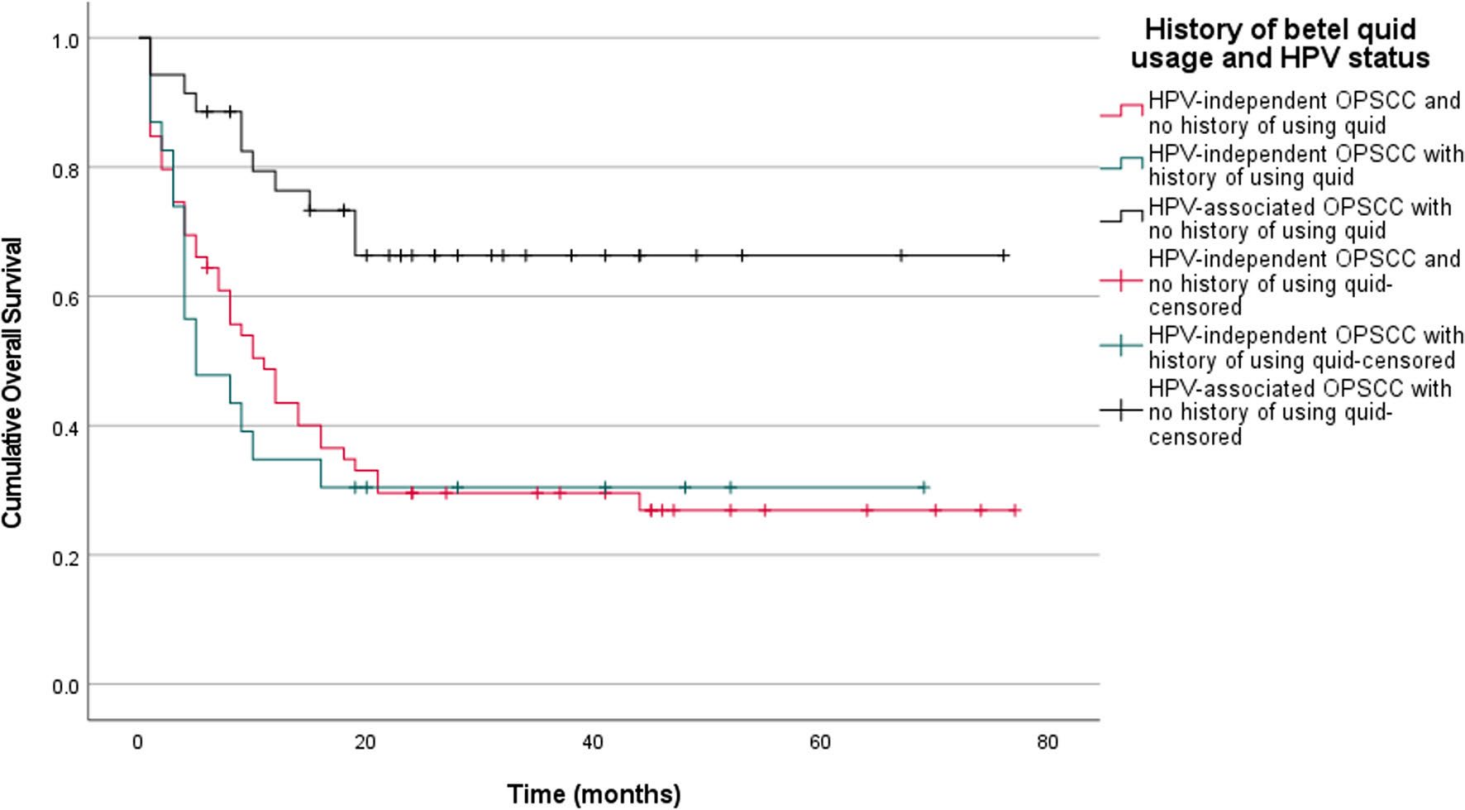
Kaplan Meier estimates of overall survival stratified according to history of betel quid use in combination with HPV status. All HPV-associated OPSCC patients were found to have never used (chewed) betel quid and had much better overall survival rates compared to the other sub-groups (Log-rank test, pooled over strata; *p* < 0.001; n = 117). However, pairwise statistical comparisons between patients with HPV-independent OPSCC who had no history of betel quid usage and those that did, showed no statistically significant difference (Log-rank test, pairwise over strata; *p* = 0.738; n = 117)

**Fig. 4:**
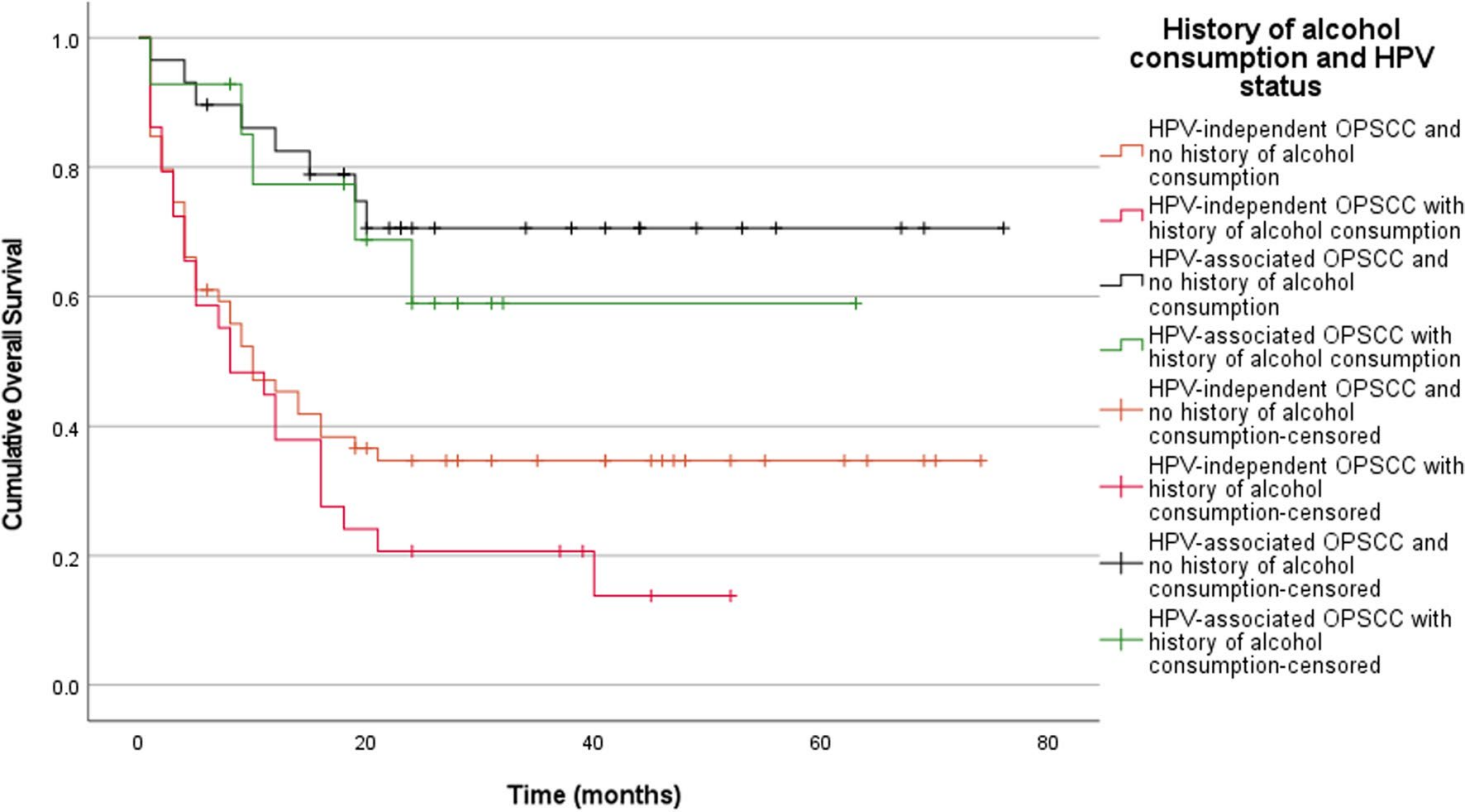
Kaplan Meier estimates of overall survival stratified according to history of alcohol consumption in combination with HPV status. Patients with HPV-associated OPSCC were found to have better overall survival rates compared to HPV-independent OPSCC patients (Log-rank test, pooled over strata; *p* < 0.001; n = 131). Pairwise statistical comparisons between patients with HPV-associated OPSCC who had no history of alcohol consumption and those that did, showed no statistically significant difference (Log-rank test, pairwise over strata; *p* = 0.612; n = 131). Similarly, pairwise statistical comparisons between patients with HPV-independent OPSCC who had no history of alcohol consumption and those that did, showed no statistically significant difference (Log-rank test, pairwise over strata; *p* = 0.222; n = 131)

Survival analysis showed that patients with HPV-associated OPSCC had a much better overall survival than patients with HPV-independent OPSCC (Fig. 1; Log rank test; *p* < 0.001). Sub-analysis on habit-related risk factors revealed that patients who were never smokers and had HPV-associated OPSCC were found to have much better overall survival when compared to all other sub-groups. Patients who had a positive smoking history (current/former) and HPV-independent OPSCC had the worst overall survival (Fig. 2; Log-rank test; *p* < 0.001; n = 146). Similarly, HPV-associated OPSCC patients with no history of betel quid usage had much better overall survival rates compared to the other sub-groups (Fig. 3; Log-rank test; *p* < 0.001; n = 117). However, the overall survival of patients with HPV-independent OPSCC with or without a history of betel quid use were rather similar (Fig. 3; Log-rank test; *p* = 0.738; n = 117). Interestingly, in the sub-analysis for alcohol consumption status, non-drinkers appeared to do slightly better than drinkers in both sub-groups, however, the findings were not statistically significant (Fig. 4; Log-rank test; *p* > 0.05; n = 131).

**Fig. 5:**
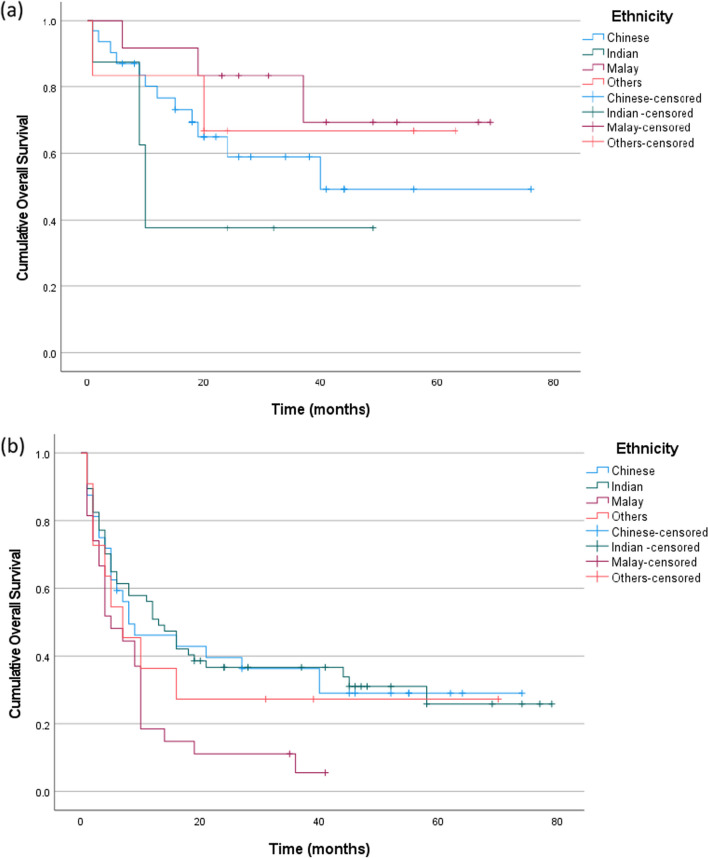
Kaplan Meier estimates of overall survival stratified according to ethnicity of patients. **a** There was no statistically significant difference in overall survival for patients with HPV-associated OPSCC of different ethnic groups (Log-rank test, pooled over strata; *p* = 0.234; n = 57); **b** Patients stratified according to ethnicity of patients. There was statistically significant difference in overall survival for patients with HPV-independent OPSCC of different ethnic groups, with patients of Malay ethnicity having poorer overall survival when compared to patients of other ethnicity (Log-rank test, pooled over strata; *p* = 0.028; n = 127)

Overall survival for patients with HPV-associated OPSCC was not influenced by ethnicity (Fig. 5; Log-rank test, pooled over strata; *p* = 0.234; n = 57), however for patients with HPV-independent OPSCC, patients of Malay ethnicity were seen to have poorer overall survival when compared to patients of other ethnicity (Fig. 5; Log-rank test, pooled over strata; *p* = 0.028; n = 127).

## Discussion

The incidence of OPSCC in Malaysia has been on an increasing trend based on the last two Malaysian National Cancer Registry Reports [14, 15]. The estimated prevalence of HPV-associated OPSCC of 31.0% from our study was slightly higher than the 25% prevalence rate found by a previous Malaysian study published in 2018 [10]. The aforementioned study looked at 60 cases of OPSCC curated over a 12-year period (2004–2015). Aside from possible increase in the HPV-associated fraction of OPSCC over the years, the vast difference in sample size between the current study and the previous study by Yap et al. could also be a contributing factor to this difference in prevalence rates.

Although the prevalence rates from our study are comparable to reported rates from Singapore and lower than that reported in other developed Asian countries such as Japan, South Korea and China, cross comparison between epidemiological studies are hampered by the criteria used to define HPV status in OPSCC [16–20]. Numerous definitions have been used to define the HPV status of OPSCC in the literature with some investigators using additional molecular methods to determine if a tumour is truly “HPV-driven” [21–27]. The prognostic utility of such dual or tiered testing methods that involve more complex molecular methods has been quite promising, though it may be difficult to roll out such assays as readily as p16^INK4a^ IHC to the general, hospital-based histopathology laboratories around the world [23–27]. However, such dual- or three-tiered testing may be appropriate in clinical trials that involve treatment de-escalation so that allocation of patients to treatment arms is done with better accuracy. Although a surrogate marker, the utilization of p16^INK4a^ IHC staining as a defining criteria for HPV-associated OPSCC has been included in the 8^th^ Edition of the AJCC cancer staging manual as well as the latest edition of the World Health Organization (WHO) reference text on Head and Neck Tumours and as such it is advisable to include assessment of p16^INK4a^ IHC in any study involving OPSCC to enable cross-comparisons [9, 12].

An interesting observation from our study is the relatively similar age at diagnosis between patients with HPV-associated OPSCC and HPV-independent OPSCC. The median values for each sub-group showed that the HPV-associated OPSCC patients were only slightly younger than the HPV-independent OPSCC patients. Though most earlier epidemiological studies on HPV-associated OPSCC have suggested that these patients are usually younger that patients with HPV-independent OPSCC patients, this trend may be shifting and HPV-associated OPSCC cases may no longer be frequently encountered in younger patients [12, 17, 21, 28–31]. This trend has serious implications as the survival advantage demonstrated in patients with HPV-associated OPSCC may be less pronounced in older patients especially those with more co-morbidities.

Although OPSCC was most frequently seen among patients of the Indian ethnic group, the vast majority of the Indian patients had HPV-independent OPSCC (57/65 cases). More than half of all the HPV-associated OPSCC patients in our study were from the Chinese ethnic group. Almost 50% (31/63 cases) of Chinese patients with OPSCC had HPV-associated OPSCC and in fact more than half of the HPV-associated OPSCC disease burden is experienced by the Chinese ethnic group. It is worth noting that when compared to the findings from the earlier study by Yap et al. [10] the proportion of HPV-associated OPSCC in both the Chinese and Indian ethnic groups appears to be increasing. This difference in prevalence rates between ethnic groups warrants further research into risk factors that may predispose to HPV-associated OPSCC such as sexual behaviour and other lifestyle-related factors. [32, 33] Sexual transmission of high-risk HPV between partners/couples has already been implicated in cases of concurrent HPV-associated OPSCC in couples [34, 35]. However, no such cases were seen in our cohort of patients. In patients with HPV-independent OPSCC, patients from the Malay ethnic group appeared to have worse overall survival when compared to other ethnic groups. This difference was not seen in patients with HPV-associated OPSCC. This may be due to other patient-related prognostic factors such as diet, habits (smoking, etc.) and other co-morbidities.

Consistent with other studies on OPSCC, our patients with HPV-associated OPSCC were shown to have better overall survival when compared to patients with HPV-independent OPSCC [12, 17, 36–38]. The prognosis of patients with OPSCC, regardless of whether HPV-associated or otherwise, appears to be adversely affected by smoking history, history of betel quid usage as well as history of alcohol consumption. Our findings are similar to that reported by others [36, 39]. As such, it is imperative to always take into account other patient-related prognostic factors such as smoking history, betel quid chewing and alcohol consumption, just to name a few. A limitation in our study is the lack of in-depth data on patient-related (such as smoking pack-years, etc.) and treatment-related (treatment provided, etc.) prognostic factors. Future studies should aim to obtain more detailed accounts of patient-related and treatment-related prognostic factors.

Another possible limitation in our study relates to the utilisation of TMAs for p16^INK4a^ IHC. The small cores sampled from the tissue block may not be completely representative of whole tumours as tumours tend to be heterogenous and may express proteins at varying levels in different regions [40]. However, evaluation of p16^INK4a^ IHC on TMAs has been performed successfully by several research groups [27, 41, 42].

## Conclusion

The findings from our study suggests that prevalence of HPV-associated OPSCC in Malaysia, though not as high as some developed countries, is however on an upward trend. HPV-associated OPSCC appears to be more frequently encountered in patients of Chinese ethnicity. The shift of HPV-associated OPSCC from “younger” to “older” patients is also of concern with regard to prognosis and burden on the healthcare system. Conventional risk-factors associated with OPSCC such as smoking, alcohol consumption and betel quid chewing should still be considered when estimating prognosis of patients with HPV-associated OPSCC as they may attenuate the positive prognostic effects of HPV status.

## Data Availability

The datasets used and/or analysed during the current study are available from the corresponding author on reasonable request.

## Abbreviations

AJCC: American Joint Committee on Cancer
FFPE: Formalin-fixed paraffin-embedded
GLOBOCAN: Global Cancer Observatory
HPV: Human papillomavirus
H&E: Haematoxylin and eosin
IARC: International Agency for Research on Cancer
IHC: Immunohistochemistry
IQR: Interquartile range
NRD: National Registration Department
OPSCC: Oropharyngeal squamous cell carcinoma
SCC: Squamous cell carcinoma
TMA: Tissue microarray
TNM: Tumour, Node, Metastasis
WHO: World Health Organization
